# Development and validation of a new scale for prediction of low back pain occurrence among nurses

**DOI:** 10.17179/excli2019-1167

**Published:** 2019-05-27

**Authors:** Mohammad Javad Jafari, Foroogh Doshman Fana Yazdi, Yadollah Mehrabi, Sakineh Rakhshanderou, Mahnaz Saremi

**Affiliations:** 1 School of Public Health and Safety, Shahid Beheshti University of Medical Sciences, Tehran, Iran

**Keywords:** low back pain, ergonomic assessment, nursing personnel, biopsychosocial factors, scale development

## Abstract

There are several scales for prediction of low back pain (LBP) occurrence, but most of them only consider occupational aspect. This study aimed to develop and validate a new biopsychosocial scale for the LBP prediction among nurses. In this mixed-method study, a scale was developed by integrating the findings from the literature review and the semi-structured interviews. The qualitative and quantitative face and content validation were then performed. The construct validation was performed based on the hypothesis testing by independent-samples t-test using the SPSS in a case study with 241 nurses. The reliability of the scale was also tested through 15-day interval test-retest reliability by Intra Class Correlation Coefficient (ICC). Then the Minimum Detectable Changes (MDC) and MDC % was calculated. The results showed that the three dimensions (occupational, psychosocial and individual), consisted of 40 items, predict LBP occurrence. Both the scale and the three sub-scales could differentiate known groups of nurses in terms of LBP. These groups were nurses: with/without LBP during the past 12 years, with a high/low occurrence of LBP, with/without co-morbidity, being female/male, with/without night shift, and with high/low repetition of loads/patients handling. The average measure ICC of the scale was 0.866 (P <0.001). The MDC95 (MDC %) was 14.86 (15.65 %). We concluded that the proposed scale is a simple and trustworthy tool which supports the multidimensional nature of LBP.

## Introduction

Low Back Pain is one of the most common disorders among workforces (Piranveyseh et al., 2016[30]; Choobineh et al., 2013[8]; Motamedzade et al., 2013[28]; Mohammadi et al., 2013[27]) especially among nurses, which can lead to a decreased productivity. The decreased quality of services delivered to the patients, medication errors, etc., also the increased rate of absenteeism, as well as, direct and indirect costs are some examples of the agents affect on the productivity (Yip, 2001[42]; Asadi et al., 2016[3]). According to several recent studies, the prevalence of LBP among nurses in Iran is much higher (over 50 %) (Asadi et al., 2016[3]; Sadeghian et al., 2014[32]; Mehrdad et al., 2010[25]) than the general population (29.3 %) (Biglarian et al., 2012[7]). It is noteworthy that it is not possible to introduce only one single agent as the cause of LBP occurrence, because of the multidimensional nature of LBP, and the variability in the tasks performed at the workplaces. From the ergonomics point of view, the treatment of LBP has been often unsuccessful (Bakker et al., 2009[5]; Deyo et al., 2014[14]) and costly. So, prevention would be a more effective approach to LBP management (Bakker et al., 2009[5]; Cohen et al., 2008[9]).

To manage LBP in the workplace more effectively, we need LBP predictive scales which consider all of the effective aspects (Koes et al., 2010[21]). Several ergonomics scales are available for LBP assessment and prediction, such as The American Conference of Governmental Industrial Hygienists method (ACGIH, 2017[2]), National Institute for Occupational Safety and Health equation (NIOSH) (Waters et al., 1993[39]), etc. (David, 2005[11]). The common weakness of these scales is that they only consider the occupational aspect without addressing other effective aspects such as psychosocial ones (David, 2005[11]). Moreover, it is believed that psychosocial risk factors influence LBP occurrence through interaction with the work environment and individual characteristics (Abedini et al., 2015[1]; Dehghany et al., 2012[13]). Therefore, it is highly recommended that the researchers pay more attention to biopsychosocial factors (Mitchell et al., 2009[26]; George et al., 2012[17]; Dunn et al., 2013[15]; Dagenais et al., 2010[10]) in designing the screening scales (Pincus et al., 2002[29]).

In a biopsychosocial model, none of the individual, occupational and psychosocial dimensions can explain LBP singly (Mitchell et al., 2009[26]), and for a comprehensive explanation of LBP occurrence, the interaction within and between these dimensions should also be considered (Marras, 2005[24]). To the best of our knowledge, no such study is available in Iran. Hence, this study is intended to develop and validate a new biopsychosocial scale for the LBP prediction among nurses. 

## Material and Methods

This mixed-method study was conducted on nursing staff from June 2017 to August 2018 in Yazd, Iran, through the qualitative and quantitative methods as follows: 

### Literature review

The LBP risk assessment models and scales, as well as, the entire body risk assessment scales in the low back section were studied. For this purpose, published papers from 1990 to 2017 were included in this study. Relevant papers were explored by searching Google Scholar, Science Direct, PubMed, NCBI, and Scopus databases. The LBP related specific terms (Low back pain, or occupational low back pain with nursing, nurses, prevalence, occurrence, predictor, incidence, prognosis, first episode, first onset, the risk factor) were used to explore all databases. Two independent researchers explored the databases to find the eligible papers. Once the qualified papers were identified, the abstract and the full-text of papers were reviewed by the researchers. The extracted factors were classified into categories according to their similarities.

### Qualitative study 

A qualitative study was carried out to confirm the agents obtained from the literature review. 

#### Participants

The participants were experienced nurses in the nursing care fields. A purposive sampling technique was employed to choose the subjects. We communicated with the hospitals to introduce the volunteer nurses in order to identify their expertise. Subsequently, the main criteria to choose the eligible experts included:

1. Having at least 5 years of job tenure in the nursing care activities
2. Having a history of Low back pain during the last year
3. Having the willingness to participate in the interview
4. Having a negative history of LBP due to a specific causation such as trauma, tumor, skeletal anomalies, spine surgery, or pregnancy during the last year.

From 43 nurses, 29 subjects who met the inclusion criteria were invited to participate in the study. Details of the study, time and location of the interview were set with each expert.

#### Data gathering

A semi-structured interview approach was employed in order to gather the experience of experts. The interview was performed based on the guide obtained from the advisers for a good interview. An interview guide was provided with several questions and supplemented with the complementary questions during the interview sessions. Each interview session lasted 15 to 30 minutes. The informed consent was obtained from the interviewees for recording the interviews. After each interview, the recorded contents were listened thoroughly and the sentences were transcribed. Subsequently, the contents were derived, coded, and classified by careful studying of the transcripts. According to the basic theory of the study, coding and classifying the extracted concepts were carried out inductively. Data saturation was obtained after analyzing the 26 interviews which continued for all 29 subjects. The extracted codes were classified into the categories based on their similarities.

### Construction of the scale 

#### Aggregating the qualitative study and literature review findings

The findings of the qualitative study and the literature review were aggregated into a draft model. We also checked the draft to remove duplicated factors. 

#### Psychometric properties of the questionnaire

##### Validity analysis

Validity means the degree to which a scale can correctly measure the target (Jafari et al., 2017[19]). The validity of the proposed questionnaire was assessed as follows: 

##### Face validity

Face validity was measured qualitatively by sending a Persian version of the questionnaire to 45 experts (5 experts at the academic level and 40 nurses) and receiving their overall conception in responding to the statement content for all of the items.

For quantitative face validity, an impact score was computed through a 5-point Likert scale, in which, the "always" scale received the score 5 and score 1 belonged to the "never" scale. To obtain the impact score, frequency (the percent of responses with the important score of 4 or 5) and item's importance (importance of each item on a 5-point Likert scale) should be calculated. Item impact score was obtained by multiplying the item's frequency and importance. The cutoff point to select the eligible items was calculated to be 1.5 (means that an item has the frequency of 50 % and the importance of 3 on the Likert scale). The values below 1.5 were removed from the questionnaire (Zamanzadeh et al., 2015[43]). 

##### Content validity

The questions in the prepared draft were categorized into the three sub-scales: individual, occupational, and psychosocial.

According to the Lawshe table (Lawshe, 1975[22]), 5 through 40 experts should be chosen based on the accessibility to the experts. In this study, fifteen experts related to each sub-scale (a total of 45 experts) were called to administer the content validation forms. Every specialist rated the 'necessity of each item' by selecting one of the three options 'essential', 'useful but not essential', or 'not essential'. Content validity ratio (CVR) for each item was calculated based on the ratings, according to Equation 1:


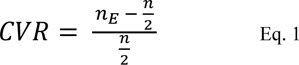


where n represents the number of raters, and n_E_, represents the number of raters who considered the item “Essential”.

Based on the Lawshe table (Lawshe, 1975[22]) the acceptable CVR score was 0.49. Therefore, the items with scores lower than the cut point 0.49 were discarded. The scale's content validity index (S-CVI) was determined by calculating the mean CVR for the total items remained in the scale (Lawshe, 1975[22]). The values higher than 0.8 were accepted (Polit and Beck, 2010[31]). The clarity and simplicity of the scale were investigated as well.

##### Construct validity

The scale was distributed between nurses of all 5 public Hospitals in Yazd province, Iran. The stratified random sampling approach was used for data sampling. It means that samples were taken from all wards, based on the nursing population occupied in each ward. The sample size was estimated to be at least 5 people per variable (Ebadi et al., 2017[16]). Thus, the nurses who met inclusion criteria, whether having or not having LBP during the past 12 years formed the sample population.

After collecting the distributed scales, the data were entered into SPSS software. It should be mentioned that the convergent and discriminant validity through explanatory factor analysis were obtained undesirable. Therefore, the explanatory factor analysis can't be a proper option to test the construct validity. In this situation, a scale should be tested by the proper hypotheses to approve the proven facts. The scores were calculated at both levels: the whole scale and the sub-scales. The validity of this scale was checked by hypothesis testing. Since the scale scores should be significantly different between the groups with different levels of LBP risk, the hypothesis testing should be able to reject the null hypothesis on the equality of means. The independent-samples t-test was used by SPSS software to compare means. The following groups were compared: the nurses with/without low back pain during the past twelve months, the nurses with high/low frequency of LBP, the nurses with/without co-morbidity, female/male nurses, and night/day shift nurses. 

##### Reliability testing

The reliability testing for the whole scale was performed using a test-retest method. The developed scale was administered by 30 nurses with a 15-day interval between test and retest. For every participant, the whole scale score was calculated at both test and re-test stage by summing the scores of all items. Then the Intra Class Correlation Coefficient (ICC) (Dagenais et al., 2010[10]) was estimated for the two scores by means of the SPSS (Version 20) in two-way mixed mode for absolute agreement. The results were interpreted based on the following criteria: 0 0.0-0.2 (low), 0.21-0.40 (fair), 0.41-0.60 (moderate), 0.61-0.80 (substantial), and 0.81-1.0 (almost perfect) (Sharif Nia et al., 2013[35]).

The absolute reliability of the scale was tested by the equations 2 and 3, respectively:


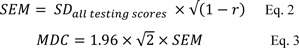


Here, SD is the standard deviation of all testing scores, and r is the coefficient of the test-retest reliability (ICC) (Lee et al., 2017[23]). 

The MDC % was also calculated by equation 4:


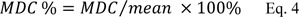


Here, mean is the mean score of all trials. An MDC % of 30 % or less was considered acceptable (Azadi et al., 2015[4]). 

## Results

Table 1(Tab. 1) illustrates the demographic information about the participants in the cross-sectional study. The participants were mostly female (80.9 %) and married (81.7 %). The mean age of the participants was 35.7 ± 6.3 (range: 25-55). The mean job tenure of the participants was 11 years, ranging from 5 to 29 with an interquartile range of 5-29.

A total of 86 variables from the literature review and 36 variables from the qualitative study were identified. After removing duplicated items, 86 items remained in draft scale.

The results of quantitative face validity indicated that "impact scores" for all of the items were higher than 1.5. Hence, all of the items remained for the following steps. Most of the experts stated that they had no difficulty in reading and understanding the questionnaire items. According to the participated nurses, a few items needed to be modified to enhance the face validity, so all of which were corrected.

Based on the Lawshe table, forty-six items were removed due to CVR scores lower than 0.49. The overall scale's content validity (S-CVI) was measured to be 0.81.

Forty items remained in the questionnaire consisted of 9 psychosocial items, 12 occupational items, and 19 individual items. The minimum acceptable sample size was obtained 220 (By considering 5 samples per each item). But a total of 241 nurses was included in this study, which returned the questionnaires. 

The null hypothesis on the equality of means of the whole scores was rejected between some known groups (Table 2(Tab. 2)). 

In addition, the null hypothesis on the equality of means of the sub-scale scores was rejected between the known groups by the individual, occupational, and psychosocial sub-scales respectively (Tables 3(Tab. 3), 4(Tab. 4), and 5(Tab. 5)).

On the reliability testing stage, the average measure ICC was 0.866 with a 95 % confidence interval from 0.687 to 0.943 (F = 7.38, P <0.001). The SEM and MDC95 were 5.47, and 15.16 respectively. The MDC % was equal to 15.97 %.

The final scale for the prediction of LBP occurrence among nurses is shown in the Appendix (Supplementary material).

## Discussion

This study is intended to develop and validate a novel scale for the prediction of LBP occurrence among nurses. The developed scale consisted of the three sub-scales including individual, occupational, and psychosocial. The structural validity demonstrated that the scale could predict the risk of LBP well, because it was able to distinguish the known groups with different levels of LBP risk. Further, the scale is able to distinguish those who had LBP in the past 12 months and those who had not experienced LBP in the recent 12 months. The former group obtained higher scores than the latter group. The scale also distinguishes the nurses with high and low frequency of LBP (the former group received a higher score). Other groups that the scale is able to differentiate are presented in the following paragraphs.

### Women versus men

The relationship between LBP and woman gender has been shown in various studies (Troussier et al., 1999[36]; Schneider et al., 2006[34]; Bejia et al., 2005[6]; Wáng et al., 2016[38]; Yang et al., 2016[40]). Wáng et al. (2018[37]) indicated that among all age groups, the prevalence of LBP is higher in women compared with men (Wáng et al., 2018[37]). Similarly, this developed scale is able to differentiate these two groups by giving higher scores of women. Wáng et al. also identified the psychological factors as one of the possible causes for the higher prevalence of LBP in women compared with men (Wáng et al., 2018[37]). In this study, it was the psychosocial sub-scale that revealed the difference in prevalence of LBP between women and men.

### Co-morbidity versus absence of co-morbidity 

Different studies have shown the association between co-morbidity and low back pain (Hestbaek et al., 2004[18]; Schneider et al., 2007[33]; de Luca et al., 2017[12]). The concurrent diseases can be musculoskeletal disorders, such as rheumatoid arthritis, osteoarthritis, and osteoporosis (Schneider et al., 2007[33]), or diseases such as diabetes, cardiovascular or pulmonary diseases (de Luca et al., 2017[12]) or headache and asthma (Hestbaek et al., 2004[18]). The scale gives higher scores in case of co-morbidity.

### Working in night shifts versus day shifts

Studies have shown the relationship between shift working and the prevalence of LBP (Zhao et al., 2012[44]; June et al., 2011[20]). In the present study, the scores were higher in nurses who worked in night shifts than those who did not.

### High frequent versus low frequent patients/loads handling 

More frequent lifting during a shift increases the likelihood of LBP occurrence. Even lifting the light loads with high frequency can contribute to the occurrence of LBP. For example, if a person lifts 11.3 Kg weight 25 times a day, the risk of acute LBP increases by 3 times (Yip, 2001[42]; Yasobant and Rajkumar, 2014[41]). The occupational sub-scale could well differentiate these two groups by giving higher scores to nurses with more frequent load/patient handling. 

The present study was an attempt to propose and validate a new scale which supports the multidimensional nature of LBP. The final scale consisted of the individual, occupational, and psychosocial dimensions. Furthermore, the proposed scale is a sim-ple, reliable and validated scale to predict LBP in nursing activities.

## Conflict of interest

The authors declare that there are no conflicts of interest.

## Acknowledgements

This paper was submitted as part of a thesis for the Ph.D. degree in Occupational Hygiene, School of Health and Safety, Shahid Beheshti University of Medical Sciences, Tehran, Iran. We deeply appreciate the guidance and collaboration of Dr. Hamid Sharif Nia from The Mazandaran University of Medical Sciences, Dr. Heidar Mohammadi from The Larestan University of Medical Sciences, and Dr. Yalda Hashempour from The Mazandaran University of Medical Sciences.

## Funding

This research received no specific grant from any funding agency in the public, commercial, or not-for-profit sectors.

## Supplementary Material

Supplementary material

## Figures and Tables

**Table 1:**
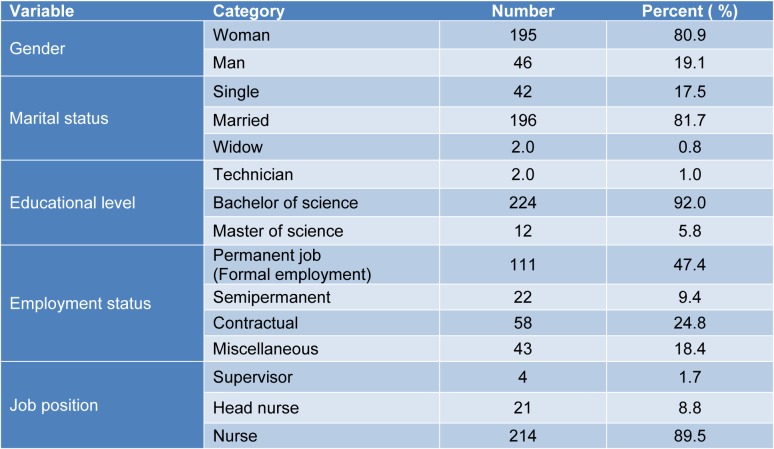
Details of the participants involved in the cross-sectional study (n=241)

**Table 2:**
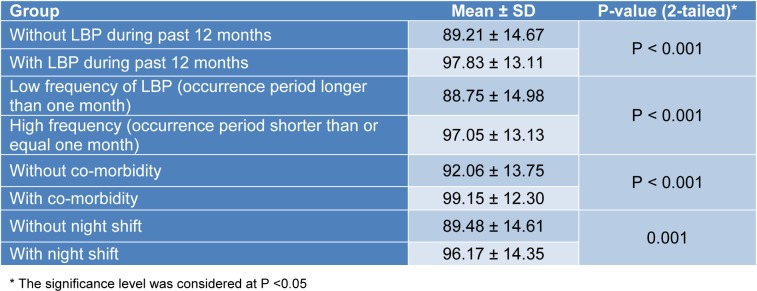
The results of the hypothesis testing with the whole scale scores

**Table 3:**
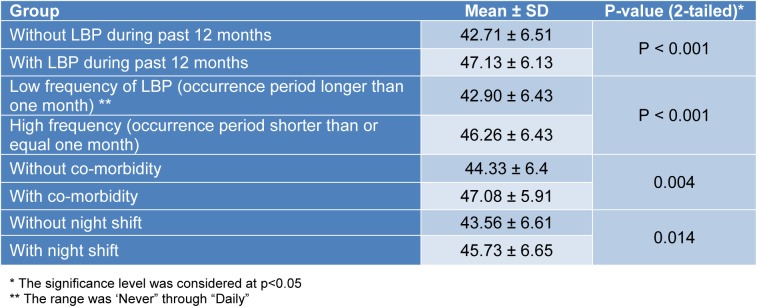
The results of the hypothesis testing with the Individual sub-scale scores

**Table 4:**
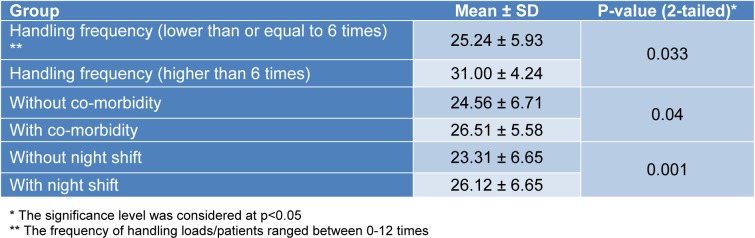
The results of the hypothesis testing with the Occupational sub-scale scores

**Table 5:**
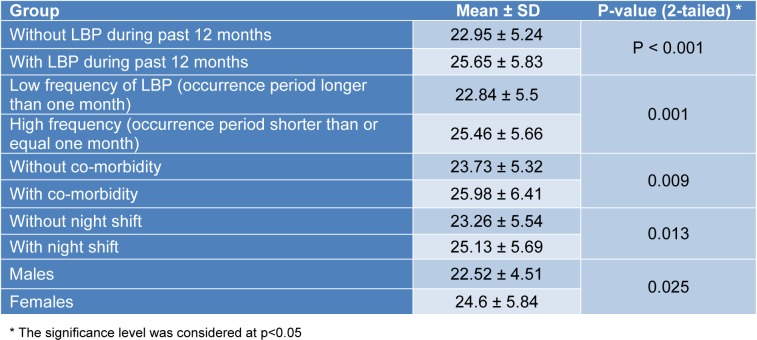
The results of the hypothesis testing with the Psychosocial sub-scale scores

## References

[1] Abedini R, Soltanzadeh A, Faghih MA, Mohammadi H, Kamalinia M, Mohraz MH (2015). Health consequences of shift-work: The case of Iranian Hospital Security Personnel. Work.

[2] ACGIH (2017). Threshold limit values for chemical substances and physical agents and biological exposure indices.

[3] Asadi P, Monsef Kasmaei V, Zia Ziabari SM, Zohrevandi B (2016). The prevalence of low back pain among nurses working in Poursina hospital in Rasht, Iran. J Emerg Pract Trauma.

[4] Azadi F, Parnianpour M, Shakeri H, Kazemnejad A, Kamrani AA, Arab A (2015). How much change in the 5-repetition sit-to-stand test is considered real in community dwelling elderly and healthy young adults. Salmand.

[5] Bakker EWP, Verhagen AP, Van Trijffel E, Lucas C, Koes BW (2009). Spinal mechanical load as a risk factor for low back pain: a systematic review of prospective cohort studies. Spine.

[6] Bejia I, Younes M, Jamila HB, Khalfallah T, Salem KB, Touzi M (2005). Prevalence and factors associated to low back pain among hospital staff. Joint Bone Spine.

[7] Biglarian A, Seifi B, Bakhshi E, Mohammad K, Rahgozar M, Karimlou M (2012). Low back pain prevalence and associated factors in iranian population: findings from the national health survey. Pain Res Treat.

[8] Choobineh A, Daneshmandi H, Aghabeigi M, Haghayegh A (2013). Prevalence of musculoskeletal symptoms among employees of Iranian petrochemical industries: October 2009 to December 2012. Int J Occup Environ Med.

[9] Cohen SP, Argoff CE, Carragee EJ (2008). Management of low back pain. BMJ.

[10] Dagenais S, Tricco AC, Haldeman S (2010). Synthesis of recommendations for the assessment and management of low back pain from recent clinical practice guidelines. Spine J.

[11] David G (2005). Ergonomic methods for assessing exposure to risk factors for work-related musculoskeletal disorders. Occup Med.

[12] de Luca KE, Parkinson L, Haldeman S, Byles JE, Blyth FJ (2017). The relationship between spinal pain and comorbidity: a cross-sectional analysis of 579 community-dwelling, older Australian women. J Manipulative Physiol Ther.

[13] Dehghany M, Rezaeeyani M, Mohammadi H, Zamanian Z (2012). An investigation of shift work disorders in security personnel of 3 hospitals of Shiraz University of Medical Sciences, 2009. Iran Occup Health.

[14] Deyo RA, Dworkin SF, Amtmann D, Andersson G, Borenstein D, Carragee E (2014). Report of the National Institutes of Health Task Force on Research Standards for Chronic Low Back Pain. J Manipulative Physiol Ther.

[15] Dunn KM, Hestbaek L, Cassidy JD (2013). Low back pain across the life course. Best Pract Res Clin Rheumatol.

[16] Ebadi A, Zarshenas L, Rakhshan M, Zareiyan A, Sharif Nia H, Mojahedi M (2017). Principles of scale development in health sciences.

[17] George SZ, Childs JD, Teyhen DS, Wu SS, Wright AC, Dugan JL (2012). Predictors of occurrence and severity of first time low back pain episodes: findings from a military inception cohort. PLoS One.

[18] Hestbaek L, Leboeuf-Yde C, Kyvik KO, Vach W, Russell MB, Skadhauge L (2004). Comorbidity with low back pain: a cross-sectional population-based survey of 12-to 22-year-olds. Spine.

[19] Jafari MJ, Eskandari D, Valipour F, Mehrabi Y, Charkhand H, Mirghotbi M (2017). Development and validation of a new safety climate scale for petrochemical industries. Work.

[20] June KJ, Cho S-H (2011). Low back pain and work-related factors among nurses in intensive care units. J Clinic Nurs.

[21] Koes BW, Van-Tulder M, Lin CWC, Macedo LG, McAuley J, Maher C (2010). An updated overview of clinical guidelines for the management of non-specific low back pain in primary care. Eur Spine J.

[22] Lawshe C (1975). A quantitative approach to content validity. Pers Psychol.

[23] Lee P, Lu WS, Liu CH, Lin HY, Hsieh CL (2017). Test–retest reliability and minimal detectable change of the D2 test of attention in patients with schizophrenia. Arch Clin Neuropsychol.

[24] Marras WS (2005). The future of research in understanding and controlling work-related low back disorders. Ergonomics.

[25] Mehrdad R, Dennerlein JT, Haghighat M, Aminian O (2010). Association between psychosocial factors and musculoskeletal symptoms among Iranian nurses. ‎Am J Ind Med.

[26] Mitchell T, O’Sullivan PB, Smith A, Burnett AF, Straker L, Thornton J (2009). Biopsychosocial factors are associated with low back pain in female nursing students: A cross-sectional study. Int J Nurs Stud.

[27] Mohammadi H, Motamedzade M, Faghih MA, Bayat H, Mohraz MH, Musavi S (2013). Manual material handling assessment among workers of Iranian casting workshops. Int J Occup Saf Ergon.

[28] Motamedzade M, Faghih MA, Golmohammadi R, Faradmal J, Mohammadi H (2013). Effects of physical and personal risk factors on sick leave due to musculoskeletal disorders. Int J Occup Saf Ergon.

[29] Pincus T, Burton AK, Vogel S, Field AP (2002). A systematic review of psychological factors as predictors of chronicity/disability in prospective cohorts of low back pain. Spine.

[30] Piranveyseh P, Motamedzade M, Osatuke K, Mohammadfam I, Moghimbeigi A, Soltanzadeh A (2016). Association between psychosocial, organizational and personal factors and prevalence of musculoskeletal disorders in office workers. Int J Occup Saf Ergon.

[31] Polit D, Beck C (2010). Essentials of nursing research. Appraising evidence for nursing practice.

[32] Sadeghian F, Hosseinzadeh S, Aliyari R (2014). Do psychological factors increase the risk for low back pain among nurses? A comparing according to cross-sectional and prospective analysis. Saf Health Work.

[33] Schneider S, Mohnen SM, Schiltenwolf M, Rau C (2007). Comorbidity of low back pain: representative outcomes of a national health study in the Federal Republic of Germany. Eur J Pain.

[34] Schneider S, Randoll D, Buchner M (2006). Why do women have back pain more than men? A representative prevalence study in the Federal Republic of Germany. Clin J Pain.

[35] Sharif Nia H, Pahlavan Sharif S, Boyle C, Yaghoobzadeh A, Tahmasebi B, Rassool GH (2013). The factor structure of the spiritual well-being scale in veterans experienced chemical weapon exposure. J Relig Health.

[36] Troussier B, Marchou-Lopez S, Pironneau S, Alais E, Grison J, Pre G (1999). Back pain and spinal alignment abnormalities in schoolchildren. Rev Rhum Engl Ed.

[37] Wáng JQ, Káplár Z, Deng M, Griffith JF, Leung JCS, Kwok AWL (2018). Thoracolumbar intervertebral disc area morphometry in elderly Chinese men and women: radiographic quantifications at baseline and changes at year-4 follow-up. Spine.

[38] Wáng YXJ, Wáng JQ, Káplár Z (2016). Increased low back pain prevalence in females than in males after menopause age: evidences based on synthetic literature review. Quant Imaging Med Surg.

[39] Waters TR, Putz-Anderson V, Garg A, Fine LJ (1993). Revised NIOSH equation for the design and evaluation of manual lifting tasks. Ergonomics.

[40] Yang H, Haldeman S, Lu ML, Baker D (2016). Low back pain prevalence and related workplace psychosocial risk factors: A study using data from the 2010 national health interview survey. J Manipulative Physiol Ther.

[41] Yasobant S, Rajkumar P (2014). Work-related musculoskeletal disorders among health care professionals: A cross-sectional assessment of risk factors in a tertiary hospital, India. Indian J Occup Environ Med.

[42] Yip Yb (2001). A study of work stress, patient handling activities and the risk of low back pain among nurses in Hong Kong. J Adv Nurs.

[43] Zamanzadeh V, Ghahramanian A, Rassouli M, Abbaszadeh A, Alavi-Majd H, Nikanfar AR (2015). Design and implementation content validity study: development of an instrument for measuring patient-centered communication. J Car Sci.

[44] Zhao I, Bogossian F, Turner C (2012). The effects of shift work and interaction between shift work and overweight/obesity on low back pain in nurses: results from a longitudinal study. J Occup Environ Med.

