# Supplementary material for: Development and validation of a new scale for prediction of low back pain occurrence among nurses
Source: EXCLI J. 2019 May 27;18:277–86. doi: 10.17179/excli2019-1167 (PMC6635730; doi:10.17179/excli2019-1167)
Supplement: Supplementary material [file EXCLI-18-277-s-001.pdf]

EXCLI Journal 2019;18:277-286 – ISSN 1611-2156
Received: February 13, 2019, accepted: March 27, 2019, published: May 27, 2019

Supplementary material to:

DEVELOPMENT AND VALIDATION OF A NEW SCALE FOR PREDICTION OF LOW BACK PAIN OCCURRENCE AMONG NURSES

Mohammad Javad Jafari, Foroogh Doshman Fana Yazdi*, Yadollah Mehrabi, Sakineh Rakhshanderou, Mahnaz Saremi

School of Public Health and Safety, Shahid Beheshti University of Medical Sciences, Tehran, Iran

* Corresponding author: School of Public Health and Safety, Shahid Beheshti University of Medical Sciences, Tehran, Iran. E-mail: forfarvardin@gmail.com, Tel: +98(2122432041), + 98(9131564257), Fax: +98(2122432037)

http://dx.doi.org/10.17179/excli2019-1167

This is an Open Access article distributed under the terms of the Creative Commons Attribution License (http://creativecommons.org/licenses/by/4.0/).

EXCLI Journal 2019;18:277-286 – ISSN 1611-2156
Received: February 13, 2019, accepted: March 27, 2019, published: May 27, 2019

Appendix: The Scale for Prediction of Low Back Pain Occurrence among nurses

The Individual Sub-scale

Row	Items	Options				
1	How many hours a week do you go walking?	Never ☐	≤1 ☐	+1-3 ☐	+3-5 ☐	>5 ☐
2	How many hours a week do you do moderate exercises like cycling, or swimming with usual speed or tennis?	Never ☐	1≤ ☐	+1-3 ☐	+3-5 ☐	>5 ☐
3	How many hours a week do you do heavy exercises like aerobic, running, rapid cycling, or rapid swimming?	Never ☐	1≤ ☐	+1-3 ☐	+3-5 ☐	>5 ☐
4	How many stairs do you climb up a day in average?	<20 ☐	20-30 ☐	30-40 ☐	40-50 ☐	>50 ☐
5	Do you wear standard shoes?	Never ☐	Hardly ever ☐	Some-times ☐	Most of the time ☐	Always ☐
6	Do you use very soft and flexible bed for sleeping?	Never ☐	Hardly ever ☐	Some-times ☐	Most of the time ☐	Always ☐
7	How many hours a day do you sit on back-restless chairs, in average?	Never ☐	≤1 hour ☐	1-2 hours ☐	2-3 hours ☐	>3 hours ☐
8	Do you have bad habits in doing your activities (quick and sudden movements, quick and sudden bending and extending, twisting, etc.)?	Never ☐	Hardly ever ☐	Some-times ☐	Most of the time ☐	Always ☐
9	Do you have enough time to accomplish all daily tasks (whether home or personal)?	Never ☐	Hardly ever ☐	Some-times ☐	Most of the time ☐	Always ☐
10	Did you have any tasks in childhood or adolescence which needed doing heavy lifting or heavy-duties?	Never ☐	≤2 years ☐	2-4 years ☐	4-6 years ☐	>6 year ☐
11	Have you had a history of low back pain?	Never ☐	Hardly ever ☐	Some-times ☐	Most of the time ☐	Always ☐

EXCLI Journal 2019;18:277-286 – ISSN 1611-2156
Received: February 13, 2019, accepted: March 27, 2019, published: May 27, 2019

Row	Items	Options				
**12**	Have you had discomfort, pain or disorder in neck, or shoulders during the past month?	Never ☐	Hardly ever ☐	Some-times ☐	Most of the time ☐	Always ☐
**13**	How many hours a day do you sit in average? (work+home+out-side)	≤4 hours ☐	4-6 hours ☐	6-8 hours ☐	8-10 hours ☐	>10 hours ☐
**14**	How many hours a day, are you standing or walking, in average (work+home+outside)	≤4 hours ☐	4-6 hours ☐	6-8 hours ☐	8-10 hours ☐	>10 hours ☐
**15**	How many hours a day, do you drive in average?	Never ☐	≤1.5 hours ☐	1.5-3 hours ☐	3-4.5 hours ☐	>4.5 hours ☐
**16**	How many hours a day you are inside motor vehicles, in an aver-age?	Never ☐	≤1.5 hours ☐	1.5-3 hours ☐	3-4.5 hours ☐	>4.5 hours ☐
**17**	How many hours a day (work+home+outside) is your neck bending angle more than 20 degrees? (the shaded area in the figure below) 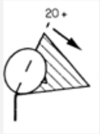	≤2 hours ☐	2-4 hours ☐	4-6 hours ☐	6-8 hours ☐	>8 hours ☐
**18**	How long do you seat on chairs with inappropriate heights?	Never ☐	≤1 hour ☐	1-2 hours ☐	2-3 hours ☐	>3 hours ☐
**19**	Do you lift loads heavier than 5 kg (boxes, babies, adults, etc.) at home or leisure time? Yes ☐ 0 No ☐ 1 If your response is No, go to the question 20.					
	Tick the type of loads and handling time a day (household or leisure activities) on the cross table.					

		≤1 hour	1-2 hours	2-3 hours	>3 hours
19-1	Boxes, cartons, and other heavy objects	2	3	4	5
19-2	Child	2	3	4	5
19-3	Adult	2	3	4	5

EXCLI Journal 2019;18:277-286 – ISSN 1611-2156
Received: February 13, 2019, accepted: March 27, 2019, published: May 27, 2019

The Occupational Sub-scale

20 Do you reposition the patients during the shift?

Yes ☐ 0 No ☐ 1 If your response is no, go on to question 21.

Tick on the cross table, the total time of repositioning during a shift, and the patient's age group.

**Duration of repositioning** / **The patient's age group**		≤0.5 hours	0.5-1 hours	1-1.5 hours	>1.5 hours
19-1	infant	2	3	4	5
19-2	child	3	4	5	5
19-3	adult	4	5	5	5

21 Do you lift and handle the patients?

Yes ☐ 0 No ☐ 1 If your response is no, go on to question 22.

Tick on the cross table, the total time of lifting and handling the patients during a shift and the patient's age group?

**Duration of lifting and handling patients** / **The patient's age group**		≤0.5 hours	0.5-1 hours	1-1.5 hours	>1.5 hours
20-1	infant	2	3	4	5
20-2	child	3	4	5	5
20-2	adult	4	5	5	5

22 Do you lift loads (other than patients) during the shift?

Yes ☐ 0 No ☐ 1 If your response is no, go on to question 23.

Tick on the cross table, the total time of load lifting during the shift and the weight of the loads? (Note: you can choose more than one cell.

**Duration of load lifting** / **Load's weight**	≤0.5 hours	0.5-1 hours	1-1.5 hours	>1.5 hours
≤5 kg	2	3	4	5
5-10 kg	3	4	5	5
10-15 kg	4	5	5	5
>15 kg	5	5	5	5

EXCLI Journal 2019;18:277-286 – ISSN 1611-2156
Received: February 13, 2019, accepted: March 27, 2019, published: May 27, 2019

**23**	Do you push or pull the patients or the objects during the shift?<br>Yes ☐ 0 No ☐ 1 If your response is no, go on to question 24.
	Tick on the cross table the total time of pulling or pushing during the shift and the load's weight?

**Duration of pushing/pulling** / **The load's weight**	≤0.5 hours	0.5-1 hours	1-1.5 hours	>1.5 hours
≤25 kg	2	3	4	5
25-35 kg	3	4	5	5
35-45 kg	4	5	5	5
>45 kg	5	5	5	5

**24**	Do you do any tasks (other than lifting, handling or pushing) during which your body be placed in a bending position?<br>Yes ☐ 0 No ☐ 1 If your response is no, go on to question 25.
	Tick on the cross table, your trunk bending angle and the total time of that during the shift.<br>(You may use the pictures as a guide to determine the bending degrees)

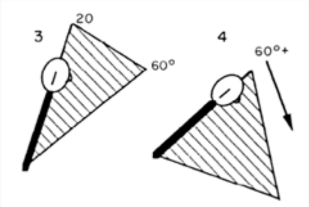

**Duration of being in bended postures** / **Trunk position**	≤0.5 hours	+0.5-1 hours	+1-1.5 hours	1.5< hours
Forward bending 21-60 degrees (Figure 3)	2	3	4	5
Forward bending ≤60 degrees (Figure 4)	3	4	5	5
Backward bending 1-20 degrees	2	3	4	5
Backward bending ≤20 degrees	3	4	5	5

**25**	Does the limited space of your work station make you uncomfortable for working or applying forces?	Never ☐	Hardly ever ☐	Sometimes ☐	Most of the time ☐	Always ☐
**26**	Are your recovery breaks (rest periods or lighter parts of work between the ponderous and tiring parts of your work) enough for refreshing?	Never ☐	Hardly ever ☐	Sometimes ☐	Most of the time ☐	Always ☐

EXCLI Journal 2019;18:277-286 – ISSN 1611-2156
Received: February 13, 2019, accepted: March 27, 2019, published: May 27, 2019

**27**	**In average, how many hours a week do you work? (Please consider the second occupation if any.)**	**≤40 hours** ☐	**40-50 hours** ☐	**50-60 hours** ☐	**60-70 hours** ☐	**>70 hours** ☐
**28**	How many 12-hour shifts (long shifts) do you have in a month?	0 ☐	1-2 ☐	3-4 ☐	5-6 ☐	>6 ☐
**29**	How many years have you been occupied in nursing?	≤5 hours ☐	5-10 years ☐	10-15 years ☐	15-20 years ☐	>20 years ☐

EXCLI Journal 2019;18:277-286 – ISSN 1611-2156
Received: February 13, 2019, accepted: March 27, 2019, published: May 27, 2019

The Psychosocial Sub-scale

**30**	Have you experienced the following symptoms during the past month?						
	30-1	I was unable to become enthusiastic about anything	Never ☐	Hardly ever ☐	Sometimes ☐	Most of the time ☐	Always ☐
	30-2	I felt down-hearted and blue	Never ☐	Hardly ever ☐	Sometimes ☐	Most of the time ☐	Always ☐
**31**	Is the relationship between you and your immediate superior a source of stress to you?		Never ☐	Hardly ever ☐	Sometimes ☐	Most of the time ☐	Always ☐
**32**	Is the relationship between you and your co-workers a source of stress to you?		Never ☐	Hardly ever ☐	Sometimes ☐	Most of the time ☐	Always ☐
**33**	If needed, can you get support and help with your work from your co-workers?		Never ☐	Hardly ever ☐	Sometimes ☐	Most of the time ☐	Always ☐
**34**	Is your job stressful?		Never ☐	Hardly ever ☐	Sometimes ☐	Most of the time ☐	Always ☐
**35**	Does your job can damage to you?		Never ☐	Hardly ever ☐	Sometimes ☐	Most of the time ☐	Always ☐
**36**	How much are you satisfied with your job?		Dissatisfied totally ☐	Dissatisfied to some extent ☐	Indifferent ☐	Satisfied to some extent ☐	Satisfied totally ☐
**37**	Is your low back pain intolerable for you?		Never ☐	Hardly ever ☐	Sometimes ☐	Most of the time ☐	Always ☐
